# Neutrophil-to-lymphocyte ratio and endometriosis: systematic review and meta-analysis

**DOI:** 10.3389/fmed.2026.1813357

**Published:** 2026-06-22

**Authors:** Mattia Dominoni, Martina Rita Pano, Adalberto Lovotti, Virginia Valeria Ferretti, Annalisa De Silvestri, Barbara Gardella

**Affiliations:** 1 Department of Clinical, Surgical, Diagnostic and Paediatric Sciences, University of Pavia, Pavia, Italy; 2 Department of Obstetrics and Gynecology, Fondazione IRCCS Policlinico San Matteo, Pavia, Italy; 3 Biometry and Clinical Epidemiology, Scientific Direction, Fondazione IRCCS Policlinico San Matteo, Pavia, Italy

**Keywords:** biomarker, CA 125, endometriosis, NLR, pelvic pain

## Abstract

**Background:**

Several biomarkers have been studied to explore the possibility of a non-invasive diagnosis of endometriosis. The aim of this review is to explore the possible role of the neutrophil-to-lymphocyte ratio (NLR) as a biomarker in the diagnosis of endometriosis.

**Methods:**

A systematic review and meta-analysis was conducted across major databases, including full-text papers in English published from 2008 to 2025. The Population, Intervention, Control, and Outcome (PICO) design approach has been used. Pooled sensibility, specificity, and positive and negative likelihood ratio were applied. A summary receiver operating characteristic curve was also plotted. The bias associated with each included study was evaluated according to the Observational Study Quality Evaluation (OSQE) method. This study is registered on PROSPERO number CRD420251004107.

**Results:**

The systematic review included seventeen retrospective studies. A total of 6,679 women were studied, of whom 3,980 had endometriosis, whereas 2,699 were in the control group (including both ovarian benign lesions and ovarian cancer). The meta-analysis conducted has shown that NLR has a sensitivity of 0.66 (0.53–0.78) and a specificity of 0.63 (0.52–0.73); in addition, it has a positive likelihood ratio of 1.79 (1.48–2.17) and a negative likelihood ratio of 0.53 (0.41–0.69).

**Conclusion:**

There are contrasting results about the non-invasive role of both CA 125 and NLR in diagnosing endometriosis. However, studies that investigated the combination of the markers showed promising results.

## Introduction

Endometriosis is characterized by the presence of endometrial tissue outside of the uterus. Its prevalence is around 10% of population (1, 2). This abnormal localization may be a cause of chronic pelvic pain and infertility, in which inflammatory molecules play an important role (3–6). Several studies have investigated the risk factors for endometriosis, suggesting that hormonal variations, such as early menarche and short periods, seem to increase the probability of developing the disease, while parity, contraceptive use, physical activity, and the assumption of omega-3 dietary fatty acids appear to be protective factors. An association between low Body Mass Index (BMI) and endometriosis has also been shown (2, 7).

The pathogenesis of endometriosis remains controversial, although there are several theories. One is implantation theory, and it supposes the presence of a reflux of endometrial tissue through the fallopian tubes that adheres to peritoneum or pelvic organs. However, not all women that have retrograde reflux have endometriosis, which suggests the presence of other factors. A second theory hypothesizes that coelomic metaplasia may be a possible source of endometriosis, through the differentiation of undifferentiated peritoneal cells into endometrial cells. Finally, a third theory speculates about a possible defect of immunity in women with endometriosis which leads to the development and the maintenance of endometriosis. In addition to these theories, recent findings also suggest a possible role of bone marrow-derived stem cells which may differentiate into endometrial cells; this can explain atypical localizations of endometriosis. Finally, genetic (4, 8) and epigenetic factors may have a role in the development of endometriosis (4, 9).

Symptoms depend on the localization of endometriosis. Some studies have revealed that the involvement of the rectum, the vagina, the sigmoid colon, and the rectovaginal septum may cause dyschezia; the involvement of the vagina, rectovaginal septum, rectum, and sigmoid colon are also responsible for dysmenorrhea; the uterosacral ligaments and parametrium localizations of endometriosis are associated with dyspareunia; finally, urinary bladder involvement is related to dysuria (10). Women with endometriosis describe pelvic pain as severe and progressive during menstrual and not-menstrual phases. Other features involve menstrual characteristics such as heavy menstrual bleeding, and irregular menstrual periods. In addition, endometriosis is associated with an increased risk for anxiety, depressive symptoms, and other psychiatric disorders (11, 12).

Endometriosis diagnosis is based on clinical suspicion derived from symptomatology, which is confirmed through imaging studies such as ultrasound (US) examination. It shows a sensibility of 35 and 61% in detecting endometrial lesions in patients with severe dysmenorrhea and endometriosis, respectively. However, it remains difficult to detect superficial lesions of the peritoneum, which are better diagnosed with laparoscopy (13). In addition to US, MRI may be used in the diagnosis of endometriosis, as second line approach, for the examination of pelvic endometriosis and in the preoperative setting for optimal pre-surgery staging (14). However, negative findings from an MRI or US do not necessarily exclude the disease. In these cases, laparoscopy may help to find lesions that are subsequently evaluated histologically (15). Several biomarkers have been studied to explore the possibility of a non-invasive diagnosis of endometriosis. For example, the role of miRNA fragments has been studied, and it has been demonstrated that they have a good sensibility and specificity in detecting endometriosis. Evaluation of a panel of cytokines, such as interleukin IL-6, IL-8, tumor necrosis factor alpha, cancer antigen 125, CA-19-9, and high-sensitivity C-reactive protein, all increase sensitivity and specificity values in the diagnosis of endometriosis compared to the use of a single biomarker (16). The aim of this review is to explore the possible role of the neutrophil-to-lymphocyte ratio as biomarker in the diagnosis of endometriosis.

## Materials and methods

### Search strategy

Articles were searched by consulting the international databases PubMed, EMBASE, and Web Of Science (Supplementary material 1). We researched the following terms and their combination: Neutrophil-to-Lymphocyte Ratio (NLR), endometriosis, CA125, and pelvic pain. The search strategy focused on studies investigating endometriosis and neutrophil-to-lymphocyte ratio (NLR). Additional clinical variables such as CA125 and pelvic pain were considered during study selection and data extraction. We have included all full-text papers in English published from 2008 to 2025 and have performed a systematic search (17). The duplicate records were removed through the Rayyan’s program which permits to identify and remove automatically the duplicate references from dataset. The Population, Intervention, Control, and Outcome (PICO) design approach was used (18, 19). The systematic review was registered in PROSPERO on 18/03/2025 with registration number CRD420251004107.

### Study selection

The articles were selected by an independent writer (MRP). Disagreements were resolved by discussion or arbitration between two senior authors (MD and BG), with both reviewers blind to each other’s assessments.

Articles regarding the role of neutrophil-to-lymphocyte ratio were included in this review. The inclusion criteria were the following: (I) articles comparing CA 125 and NLR between patients with endometriosis and patients with gynecological cancer; (II) articles comparing CA 125 and NLR among the endometriosis group, the benign ovarian tumor group, and healthy controls; (III) articles comparing mean values of CA 125 and NLR; (IV) articles comparing the sensitivity and specificity of CA 125 and NLR; (V) articles exploring the association between NLR and endometriosis-related symptoms; (VI) articles investigating differences in CA 125 and NLR values between fertile and infertile women with endometriosis; and (VII) articles comparing Visual Analog Scale (VAS) scores, CA 125 levels, and NLR. The exclusion criteria were the following: (I) publications written in a language other than English; (II) articles that included the acronym NLR but with a meaning other than neutrophil-to-lymphocyte ratio; (III) articles without full-text availability; and (IV) studies that included NLR but out of the aim of this review.

In the meta-analysis we included only studies that reported specificity and sensitivity values of NLR to assess the role of this marker in the non-invasive diagnosis of endometriosis.

### Data collection

Two authors controlled the extraction of data, and they worked independently.

### Risk of bias (RoB)

A version of the Newcastle-Ottawa Scale (NOS), which was first created for cohort and case–control studies, was used to assess the risk of bias (RoB) of cross-sectional research. The new instrument (called “NOS-xs”) uses a nine-star grading system to evaluate six distinct elements in three primary domains: (i) confounding factors, (ii) exposure and outcome evaluation, and (iii) study sample selection. Studies are classified as having high (0–3 stars), moderate (4–6 stars), or low (7–9 stars) RoB based on the quantity of awarded stars using the Newcastle-Ottawa Scale adapted for cross-sectional studies (Carra MC, 2025). Two investigators (MD and MRP) separately carried out quality assessments, and differences were settled by talking with the other authors until an agreement was achieved. RoB was evaluated only for studies included in the meta-analysis. The results were included in Supplementary material 2.

### Statistical analysis

Categorical data are reported as counts and percentages, whereas continuous variables are represented as mean ± standard deviation or as median (interquartile range [IQR]).

Diagnostic accuracy was analyzed using a bivariate random-effects model, which was specifically designed to jointly model sensitivity and specificity while accounting for their correlation and between-study heterogeneity induced by differing thresholds. A summary receiver operating characteristic (SROC) curve was also plotted. Heterogeneity was assessed by *I*^2^ index for sensitivity and specificity. A leave-one-out sensitivity analysis was performed to assess the influence of each individual study on the pooled estimates by iteratively omitting one study at a time and recomputing the summary sensitivity and specificity. This approach was used to evaluate the robustness of the meta-analytic results and to identify potential outlying studies. Publication bias was evaluated graphically and assessed using Deeks’ funnel plot asymmetry test. A *p*-value <0.10 was considered indicative of significant funnel plot asymmetry and potential small-study effects.

## Results

### Study selection

**Figure 1:**
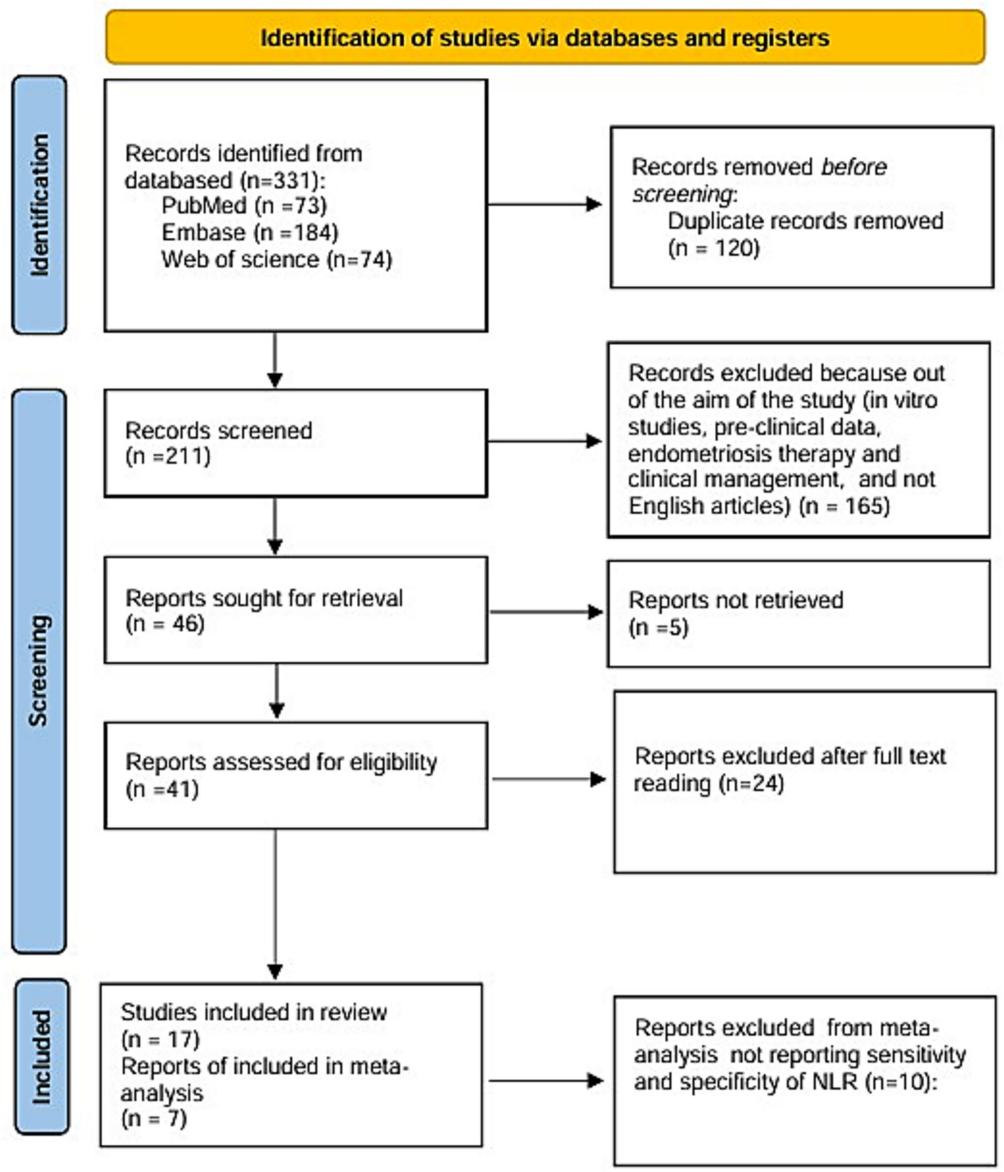
PRISMA flow diagram of the study selection process.

The research included 331 articles from the consulted databases, leaving 211 records after the removal of duplicates (120 papers). The evaluation of papers permits to select 41 eligible reports, eliminating 170 papers which do not meet the inclusion criteria or were not available. At the end, after the text analysis, only 17 were chosen for this review (Figure 1). The results are summarized in Table 1. In addition, the meta-analysis was conducted on seven articles (Table 2).

**Table 1:** Main results of included studies.

**Study and year**	**Origin of study**	**Patients enrolled**	**Age**	**Therapy for endometriosis**	**Therapy for pain**
Chen et al. (2019) (31)	China	49 women: ovarian cancer group 192 women: ovarian endometriosis group	Mean age in ovarian cancer group: 51.5 ± 10.4 Mean age in endometriosis group: 35.0 ± 7.3 *p* < 0.001	No hormone therapy in the previous 3 months	No information available
Cho et al. (2008) (22)	South Korea	231 women: endometriosis group 145 women: benign ovarian tumor group 384 women: healthy control group	Overall mean age: 33.3 ± 7.3 years Mean age in endometriosis group: 32.6 ± 7.35 years Mean age in benign ovarian tumor group: 34.2 ± 8.9 years Mean age in the healthy control group: 33.9 ± 5.7 years *p* = 104	No information available	No information available
Ding et al. (2019) (32)	China	226 women: ovarian endometrioma group 210 women: benign ovarian cyst group 112 women: control group (who underwent tubal anastomosis)	Mean age ovarian endometrioma group: 35.7 ± 0.4 Mean age benign ovarian cyst group: 35.9 ± 0.4 Mean age control group: 35.8 ± 0.5	No steroid hormone therapy in the 6 months before surgery	No information available
Dominoni et al. (2024) (21)	Italy	73 women: endometriosis group 11 patients (15.3%) stage I 17 patients (23.6%) stage II 19 patients (26.4%) stage III 25 patients (34.7%) stage IV	Overall mean age: 38 ± 7 years	Estroprogestins: 14 (19.2%) Progestogen-Only Pill: 24 (32.9%) Gonadotropin-Releasing Hormone agonists: 2 (2.7%)	No information available
Jing et al. (2020) (33)	China	662 women: endometriosis group 61 stage I-II 601 stage III-IV 83 women: benign ovarian tumors group 45 women: healthy control group	Overall mean age: 34.94 ± 9.05 years Mean age of endometriosis group: 34.08 ± 7.59 Mean age of benign ovarian tumor group: 33.29 ± 12.59 years (*p* = 0.288)	No information available	No information available
Khashchenko et al. (2023) (24)	Russia	32 patients with a confirmed diagnosis of peritoneal endometriosis	Girls aged 13–17 at diagnosis	Dienogest 2 mg daily for 1 year	Non-steroidal anti inflammatory drugs
Kim et al. (2014) (23)	South Korea	419 patients who underwent elective surgery for ovarian endometrioma 230 patients: stage IV endometriosis group 189 patients: stage III endometriosis group	Mean age in stage III endometriosis group: 33.8 ± 6.8 years Mean age in stage IV endometriosis group: 34.7 ± 7.0 years P = NS	No information available	No information available
Lin et al. (2021) (34)	China	217 infertile women with ovarian endometrioma: 90.8% stage III-IV 9.2% stage I-II	Overall mean age: 30.9 years Mean age of pregnancy success group: 30.2 years Mean age of pregnancy failure group: 31.7 years *p* = 0.006	GnRHa administration in pregnancy success group: 74 patients (72.5%) GnRHa administration in pregnancy failure group 85 (73.9) *p* = 821	No anti-inflammatory drugs during the previous 6 months
Tokmak et al. (2016) (25)	Turkey	467 patients: endometrioma group 340 patients: control group (benign ovarian cysts)	Mean age of endometrioma group: 33.7 ± 8.4 years Mean age of control group: 33.9 ± 11.6 years p = 0 0.303	No history of hormonal therapy for endometriosis	No information available
Yang et al. (2013) (35)	China	197 patients: endometriosis group 102 patients: benign ovarian tumors group 112 patients: control group	Overall mean age: 32.58 ± 6.37 years	No information available	No information available
Yavuzcan et al. (2013) (26)	Turkey	33 patients: endometrioma (OMA) group 28 patients: non-endometrioma group (benign adnexal mass other than OMA) 33 patients: control group (healthy patients who underwent tubal ligation)	Overall mean age: 36.21 ± 8.37 years Mean age in endometrioma group: 34.7 ± 9.0 years Mean age in non-endometrioma group: 37.6 ± 9.9 years Mean age in control group: 36.2 ± 8.3 years *p* = 0.403	No previous therapy for endometriosis	No information available
Gorun et al. (2024) (29)	Romania	Endometriosis pelvic pain group: 169 patients Endometriosis no pelvic pain group: 38 patients	Overall median age: 33 years Median age in pelvic pain group: 33 years Median age in no pelvic pain group: 32.5 years P=NS	No information available	No recent use of anti-inflammatory drugs
Kayacik et al. (2023) (27)	Turkey	Endometriosis group: 122 patients Control group: 145 patients	Mean age of the endometriosis group: 34.84 ± 6.75 years Mean age of the control group: 34.09 ± 6.94 years	No information available	No information available
Kedzia et al. (2023) (30)	Poland	Endometriosis group: 23 patients Control group: 10 healthy patients	No information available	No contraceptives in the last 12 months.	No information available
Ottolina et al. (2020) (20)	Italy	Endometriosis group: 324 patients 214 with endometrioma 69 with deep infiltrating endometriosis 61 with superficial peritoneal endometriosis Control group: 248 patients with other benign gynecological disease	Mean age of endometriosis group: 33.53 ± 5.51 years Mean age of control group: 31.15 ± 7.9 years *p* = 0.001	No hormonal therapy at the time of surgery Hormonal therapy before surgery: 128 women (39.5%) 64 women (25.8%) *p* = 0.43	No information available
Turgut et al. (2019) (28)	Turkey	Endometriosis group: 121 patients Control group: 136 patients	Median age in endometriosis group: 39 years Median age in control group: 38 years	No use of hormonal therapy	No use of NSAIDs
Zhou et al. (2024) (36)	China	Endometriosis group: 434 patients 237 with endometrioma 130 with adenomyosis 67 with both endometriomand adenomyosis Control group: 517 healthy patients	Overall mean age: 36 ± 6.4 years	No information available	No information available

**Table 2:** Main results of studies evaluating CA 125 and NLR.

**Study and year**	**Study design**	**NLR value**	**CA125 value**	**Pain**	**Relapse**
Chen et al. (2019) (31)	Retrospective study	Mean NLR value in ovarian cancer group: 3.23 ± 1.53 Mean NLR value in endometriosis group: 2.09 ± 1.31 (*p* < 0.001) NLR sensitivity: 92.6% NLR specificity: 79.2%	Mean log(CA125) in ovarian cancer group: 5.88 ± 1.88 Mean log(CA125) in endometriosis group: 4.05 ± 0.82 (*p* < 0.001) CA-125 specificity: 96.3% CA-125 sensitivity: 81.3%	No information available	No information available
Cho et al. (2008) (22).	Retrospective study	NLR sensitivity in the diagnosis of endometriosis: 59.7% NLR specificity in the diagnosis of endometriosis: 60.1% NLR was more increased in the endometriosis group than in the benign tumor group (*p* = 0.037) and the control group (*p* < 0.001), with significant difference between the benign tumor group and the control group (*p* = 0.044) No significant correlation was found between the stage of the disease and NLR	CA 125 sensitivity in the diagnosis of endometriosis: 55.8% CA 125 specificity: 92.8% Mean serum CA-125 levels were significantly higher in the endometriosis group than the benign tumor group and the control group (*p* < 0.001) Significant correlations were noted between the stage of the disease and serum CA-125 (*p* < 0.001).	No information available	No information available
Ding et al. (2019) (32)	Retrospective study	Mean NLR level in ovarian endometrioma group: 2.56 ± 0.07 Mean NLR level in cyst group 2.34 ± 0.07 *p* < 0.005 Sensitivity in diagnosing of OMA: 77% Specificity in diagnosing of OMA: 39.5%	Mean CA-125 level in ovarian endometrioma group: 80.0 ± 7.1 Mean CA-125 level in cyst group: 19.2 ± 0.8 *p* < 0.0001 Sensitivity in the endometriosis diagnosis: 82.3% Specificity in the endometriosis diagnosis: 90.0%	Dysmenorrhea in ovarian endometrioma group: 128 (56.6%) Dysmenorrhea in benign ovarian cysts group: 26 (12.4%) Dysmenorrhea in control group: 8 (7.1%) *p* < 0.0001	No information available
Dominoni et al. (2024) (21)	Retrospective study	NLR ≤ 2.62 (*N* = 37 patients) NLR > 2.62 (*N* = 36 patients)	Ca125 > =35 mL/L: 17 (23.3%) Ca125 < 35 mL/L: 56 (76.7%)	Women with dysmenorrhea: 32 (43.8%) Women with chronic pelvic pain: 26 (35.6%)	No information available
Jing et al. (2020) (33)	Retrospective study	NLR in I-II stage: 1.95 ± 1.12 NLR in III-IV stage: 2.37 ± 1.69 *p* < 0.05 NLR specificity: 80.20% NLR sensitivity: 32.90%	CA 125 in I-II stage: 17.28 ± 5.02 UI/ml CA 125 in III-IV stage: 82.71 ± 92.06 UI/ml p < 0.05 CA 125 specificity: 85.40% CA 125 sensitivity: 80.60%	Pain = 474 patients	No information available
Khashchenko et al. (2023) (24)	Retrospective longitudinal observational cohort study.	NLR level before therapy: 2.49 ± 1.20 NLR level after therapy: 1.56 ± 0.68 (*p* < 0.001)	CA-125 level before therapy: 26.74 ± 21.56 UI/ml CA-125 level after therapy: 12.05 ± 5.82 UI/ml (*p* < 0.001)	VAS score before therapy: 8.3 ± 1.6 VAS score after therapy: 1.7 ± 2.1 (*p* < 0.001) Persistent to NSAIDs dysmenorrhea before therapy: 96.9% Persistent to NSAIDs dysmenorrhea after therapy: 6.3% (*p* < 0.001)	No information available
Kim et al. (2014) (23)	Retrospective study	Linear correlation coefficient between NLR and endometriosis score: *r* = 0.02 (*p* value NS)	CA125 in stage III endometriosis group: 54.2 ± 87.2 (8–710) UI/ml CA125 values in stage IV endometriosis group: 67.7 ± 70.4 (10–580) UI/ml (*p*-value NS) Linear correlation coefficient between Ca125 and NLR: *r* = 0.13 (*p*-value NS)	Dysmenorrhea in stage III endometriosis group: 99 (52.4%) Dysmenorrhea in stage IV endometriosis group: 154 (67%) P = NS	No information available
Lin et al. (2021) (34)	Retrospective study	NLR low group (<1.4): 97 patients NLR high group (> 1.4): 120 patients Mean NLR value in pregnancy success group: 1.9 (1.6%) NLR mean value in patients with pregnancy failure: 1.5 (0.9%) *p* = 0.023	Overall CA 125 median value: 41.1 (27.0–68.7) UI/ml CA 125 median value in pregnancy success group: 38.3 (25.4–65.6) UI/ml CA 125 median value pregnancy failure group: 44.8 (28.5–73.5) UI/ml *p* = 0.493	Overall patients with dysmenorrhea: 139 (64.1%) Patients with dysmenorrhea in pregnancy success group: 63 (61.8%) Patients with dysmenorrhea and pregnancy failure: 76 (66.1%) *p* = 0.508	No information available
Tokmak et al. (2016) (25)	Retrospective comparative study	NLR mean value in endometrioma group: 2.8 ± 2.0 NLR mean value in the control group: 1.7 ± 0.5 *p* < 0.05 NLR diagnostic sensitivity: 70% NLR diagnostic specificity: 74%	CA 125 mean value in the endometrioma group: 45 (3.7–600) UI/ml CA 125 mean value in the control group: 12.8 (2.6–153) UI/ml *p* < 0.05 Diagnostic sensitivity of CA-125: 75% Diagnostic specificity for CA-125: 81%	Dysmenorrhea in endometrioma group: 157 (33.6%) Dysmenorrhea in control group: 91 (26.8%) *p* = 0.037 Chronic pelvic pain in endometrioma group: 257 (55%) Chronic pelvic pain in control group: 201 (59.1%) *p* = 0.247	Endometrioma group: 21.4% Control group: 8.5% *p* < 0.001
Yang et al. (2013) (35)	Retrospective study	Mean NLR value in the endometriosis group: 2.29 (2.13–2.47) Mean NLR value in the benign tumors group: 1.93 (1.80–2.06) *p* = 0.004 Mean NLR value in the healthy control group: 1.69 (1.59–1.78) *p* = 0.0001 NLR diagnostic sensitivity: 57.9% NLR diagnostic specificity: 65.2%	CA 125 in the endometriosis group: 93.49 (60.87–126.10) UI/ml CA 125 in the benign tumors group: 20.17 (18.29–22.06) UI/ml *p* = 0.001 CA 125 in the healthy control group: 16.50 (15.28–17.71) UI/ml *p* = 0.0001 Diagnostic sensitivity of CA-125: 71.6% Diagnostic specificity for CA-125: 99.1%	No information available	No information available
Yavuzcan et al. (2013) (26)	Retrospective study	NLR in the endometrioma group (stage III-IV): 2.40 ± 2.04 NLR in the non-endometrioma group: 2.51 ± 1.37 NLR in the control group: 2.11 ± 0.86 *p* = 0.555	Mean Ca-125 level in the endometrioma group: 50.8 ± 46.7 UI/ml Mean Ca-125 in the non-endometrioma group: 22.4 ± 25.3 UI/ml *p* = 0.006	Patients with dysmenorrhea: 17 (18.1%)	No information available
Gorun et al. (29)	Retrospective cross-sectional study	Mean NLR value in patients with pelvic pain: 2.01 Mean NLR value in patients without pelvic pain: 1.50 *p* = 0.011 Sensitivity in detecting pelvic pain: 59% Specificity in detecting pelvic pain: 71%	No information available	Pelvic pain: 169 patients (81.6%)	No information available
Kayacik et al. (27)	Retrospective study	Mean NLR level in endometriosis group: 3.58 ± 4.042 Mean NLR level in control group: 2.84 ± 1.75 *p* = 0.634 NLR sensitivity in diagnosis of stage III-IV of endometriosis: 32.9%	Mean Ca 125 level in endometriosis group: 82.19 ± 178.51 UI/ml Mean Ca125 level in control group: 25.81 ± 35.62 UI/ml p < 0.001 Ca125 sensitivity in diagnosis of stage III-IV of endometriosis: 64.7% Ca125 specificity in diagnosis of stage III-IV of endometriosis: 78.6%	No information available	No information available
Kedzia et al. (2023) (30)	Pilot study	NLR in control group: 1.66 NLR in endometriosis group: 4.79 *P* = 0.0001	No information available	No information available	No information available
Ottolina et al. (2020) (20)	Retrospective study	NLR in control group: 2.05 ± 1.5 NLR in endometriosis group: 2.21 ± 0.95 *p* = 0.13	No information available	No information available	No information available
Turgut et al. (2019) (28)	Retrospective study	Median NLR value in control group: 1.70 Median NLR value in endometriosis group: 2.18 *p* < 0.001 NLR sensitivity: 87.6% NLR specificity: 44.8%	Ca125 in control group: - Ca125 in endometriosis group: 48 U/mL	Chronic pelvic pain: 39 patients (32%) Dysmenorrhea: 10 patients (8%)	No information available
Zhou et al. (2024) (36)	Retrospective study	Median NLR in control group: 1.77 Median NLR in endometriosis group: 2.83 *p* < 0.0001 NLR sensitivity: 70% NLR specificity: 67%	No information available	No information available	No information available

OMA: ovarian endometrioma; NS: not significant.

### Population

This review included 17 retrospective studies published between 2008 and 2024 (Table 2). A total of 6,679 women were studied, of whom 3,980 had endometriosis, whereas 2,699 were in the control group (including both ovarian benign lesions and ovarian cancer). Two trials were conducted in Italy (20, 21), two in South Korea (22, 23), one in Russia (24), four in Turkey (25–28), one in Romania (29), one in Poland (30), and six in China (31–36).

### Intervention

We investigated the role of NLR in diagnosing endometriosis.

### Comparison

One study compared NLR’s and CA125’s diagnostic values between women with ovarian cancer and women with endometriosis (31). Four studies compared the diagnostic value of NLR and CA12T between an endometriosis group, benign ovarian tumor group, and healthy control group (22, 32, 33, 35) and five studies compared the values between an endometriosis group and a control group (20, 26, 27, 30, 36). One study explored the association between NLR, CA125, and endometriosis (25) and one explored NLR, CA125, and the stage of endometriosis (23).

Two articles studied the correlation between NLR and endometriosis-related symptoms (21), in particular pelvic pain (29).

One study also investigated the differences in CA125 and NLR values in fertile and infertile patients with endometriosis and between endometriosis and benign ovarian tumor group (33).

One study explored the role of NLR in differentiating between patients with endometrioma, cysts other than endometrioma, and controls in women with a pre-operative diagnosis of infertility (26). NLR has also been compared regarding its predictive value in natural pregnancy outcome after cystectomy for endometrioma (34). Another study compared VAS scores, CA 125 levels, and NLR levels before and after therapy with dienogest (24).

### Outcomes

Chen et al. showed that both CA 125 and NLR were higher in the ovarian cancer group than in the endometriosis group; however, the sensitivity and specificity of CA 125 were higher than those of NLR. We have no information about pain or relapses after surgery in this study (31).

Cho et al. showed that CA 125 levels and NLR mean values were both increased in the endometriosis group. In addition, CA 125 did not differ between benign the ovarian tumor group and healthy control, whereas NLR was different between the two groups. Regarding the sensitivity and specificity, NLR had lower specificity but higher sensitivity than Ca 125 in the diagnosis of endometriosis. Specifically, CA 125 has low sensitivity in detecting minimal and mild disease, whereas the mean NLR was more able to detect patients with minimal to-mild endometriosis. Combined markers showed better diagnostic value in all stages of endometriosis, with a sensitivity of 69.3% and a specificity of 83.9% (22).

Ding et al. found that both CA 125 and NLR were higher in the ovarian endometrioma group than in benign cysts group and in control group. CA 125 showed higher sensitivity and specificity in diagnosis of endometriosis than NLR; however, the combination marker had better sensitivity (32).

Dominoni et al. discovered a possible association between NLR and chronic pelvic pain in patients with histologically confirmed endometriosis, even if it was not statistically significant. This association is stronger in patients without previous therapy (21).

Jing et al. found that CA125 had better sensitivity and specificity than NLR in the differential diagnosis between endometriosis and benign ovarian tumors. In addition, they found that CA125 and NLR were both higher in endometriosis groups than in benign ovarian tumors groups; whereas these markers were both lower in endometriosis infertile groups than in endometriosis fertile groups. Finally, both markers were significantly higher in moderate and severe stages of disease. Lastly, CA 125 was higher in patients who experienced pain than in those who had no pain (33).

Khashchenko et al. evaluated the differences of NLR and CA 125 values before and after medical therapy for endometriosis, finding that their values were decreased after treatment (24).

In the study by Kim et al., no association was found between CA 125 and NLR levels and endometriosis stage III or IV (23).

Lin et al. studied the role of NLR in predicting natural pregnancy success after surgery for endometrioma removal, with the important finding that higher NLR values were associated with better natural pregnancy outcomes (34).

Tokmak et al. demonstrated that both Ca 125 and NLR were higher in women with endometrioma cysts than in women who had benign ovarian cysts. In addition, patients with endometriosis showed higher risk of dysmenorrhea, but there were no differences in chronic pelvic pain (25).

Yang et al. showed that mean Ca-125 level was higher in the endometrioma group than in the benign tumors group and in the control group. Also, NLR values were higher in the endometrioma group. Ca 125 had better sensitivity and specificity than NLR but the best sensitivity is represented by the combination marker (35).

Yavuzcan et al. found a mean Ca 125 level higher in the endometrioma group than in the non-endometrioma group. However, they found no difference among NLR values in the endometrioma, non-endometrioma, and control groups. All patients had a preoperative diagnosis of infertility (26).

Gorun et al. showed that patients with endometriosis and pelvic pain had higher values of NLR than those without pelvic pain. In addition, there was no correlation between the localization of endometriosis lesions and NLR values but a weak positive correlation was found between endometriosis stage and NLR (29).

Kayacik et al. showed that only Ca125 was higher between endometriosis patients (stages III-IV) and controls, while the NLR difference was not statistically significant, but we have to consider that the endometriosis group was composed of patients with moderate and severe endometriosis. In addition, Ca 125 had better sensitivity than NLR (27).

Kedzia et al. demonstrated that NLR was three times higher in an endometriosis group than in a healthy control group (30).

Ottolina et al. found that there was no difference between endometriosis patients and controls in NLR values. Moreover, they also found that there was no significant difference in NLR in patients with endometriosis stage 1–2 and 3–4 and controls and between different phenotypes of endometriosis. It is important to emphasize that the control group included healthy controls and women with benign gynecological conditions (20). Turgut et al. have compared 121 patients with endometriosis and 136 healthy patients demonstrating that NLR was higher in endometriosis group than in control group. However, a significant difference in NLR between stage I-II and III-IV of endometriosis was not found, but Ca 125 was higher in patients with stage III-IV of endometriosis than those with stage I-II. In addition, the sensitivity of Ca 125 was lower but its specificity in diagnosing endometriosis was higher than NLR (28).

Zhou et al. included a total of 434 women with endometriosis (237 with endometrioma, 130 with adenomiosys, and 67 with both endometrioma and adenomyosis) and they showed that NLR was higher in the endometriosis group than in control group. In addition, comparing women with adenomyosis and those with both endometrioma and adenomyosis, all had higher values of NLR than those with endometrioma only (36).

### Meta-analysis outcomes

The meta-analysis conducted has shown that NLR has a sensitivity of 0.66 (0.53–0.78) and a specificity of 0.63 (0.52–0.73); in addition, it has a positive likelihood ratio of 1.79 (1.48–2.17) and a negative likelihood ratio of 0.53 (0.41–0.69) (Table 3). Substantial heterogeneity was observed for both sensitivity (*I*^2^ = 96.3%) and specificity (*I*^2^ = 92.1%). A strong negative correlation between sensitivity and specificity (*r* = −0.81, *p* < 0.001) suggested the presence of a threshold effect.

**Table 3:** Statistical data from meta-analysis.

	Coef.	[95% Conf. Interval]	
Se	0.6651732	0.5307087	0.7772789
Sp	0.6293546	0.517698	0.7287078
LR+	1.794635	1.485734	2.167761
LR–	0.5320161	0.4068152	0.6957487
1/LR–	1.879642	1.437301	2.458119

se: sensitivity; Sp: specificity; LR+: positive likelihood ratio; LR–: negative likelihood ratio.

**Figure 2:**
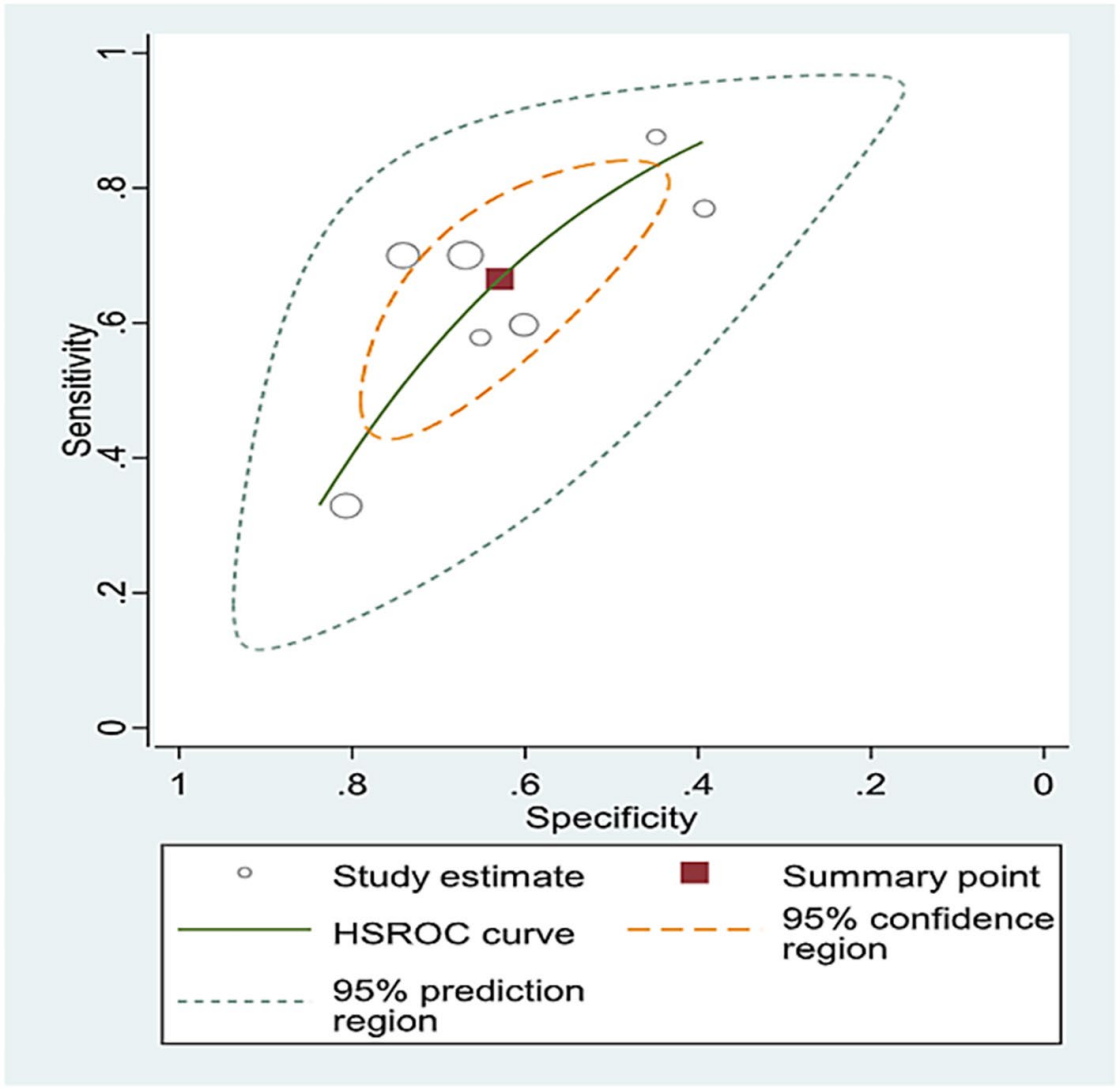
Summary receiver operating characteristic (SROC) curve.

The SROC demonstrated a low diagnostic value, as shown in Figure 2.

The leave-one-out sensitivity analysis confirmed the robustness of the pooled results, with sensitivity ranging from 0.62 to 0.71 and specificity from 0.59 to 0.66 across all iterations, indicating that no single study substantially influenced the overall estimates.

**Figure 3:**
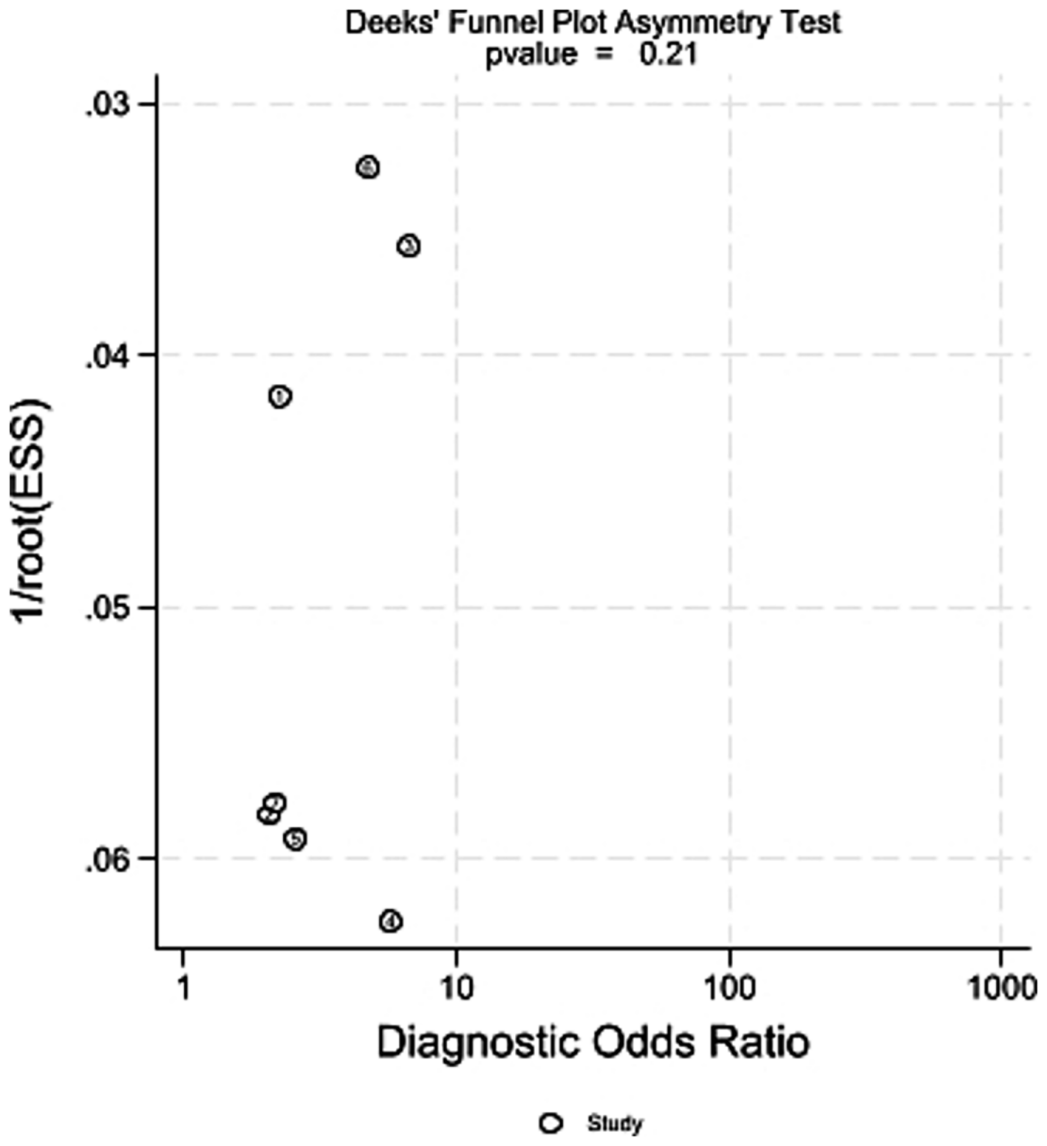
Deeks’ funnel plot.

The Deeks’ funnel plot did not show marked asymmetry, and the asymmetry test was not statistically significant (*p* = 0.21), suggesting no evidence of publication bias (Figure 3).

## Discussion

Multiple studies have tried to assess methods to diagnose endometriosis non-invasively, such as investigating the role of microRNA in differentiating endometriosis from other gynecological conditions (37). Other biomarkers have been studied, such as CA19.9 and intercellular adhesion molecule-1 (ICAM-1), however these may be increased also in benign conditions. The same issue can be found with inflammatory cytokines (38). It has been demonstrated that CA125 values in endometriosis patients are higher than in healthy population (39). In this study, we have investigated if NLR may be a possible biomarker in non-invasive diagnosis of endometriosis. It is well known that neutrophils in healthy endometrium play a role in repair and in regulating cyclic vascular proliferation. It has been also demonstrated that, in patients with endometriosis, neutrophil counts in peritoneal fluid are increased, maybe due to the augmented concentration of chemoattractants secreted by epithelial cells (4, 26).

Studies on the pathogenesis of endometriosis have investigated the alterations in T cell populations, finding that Th2 lymphocytes are more represented, while Th1 lymphocytes are suppressed, and this imbalance maintains the development of endometriosis. In addition, regulatory T cells seem to be increased in the peritoneal fluid and decreased in the peripheral blood; such an alteration may be responsible for the development of autoimmune reactions and the suppression of local cellular immune response (4). For these reasons, the studies investigated reported an increase in neutrophil count and a decrease in lymphocyte count resulting in higher NLR. The relationship between endometriosis and NLR has long been investigated (40).

In our review, we found that NLR levels were not different between patients with endometriosis, healthy patients, and women with other benign adnexal masses (26), as confirmed also by Ottolina et al. (20). Kim et al. found that NLR was not different between stage III and stage IV of endometriosis patients or between stage III-IV of endometriosis and controls (23, 27).

On the contrary, Yang et al. demonstrated that NLR was higher in endometriosis patients than in patients with benign ovarian tumor and control group (35). These findings were also confirmed by Tokmak et al. (25), by Jing et al. (33), by Ding et al. (32), and by Cho et al. (22).

Moreover, considering women with ovarian cancer (not necessarily benign adnexal masses) and women with endometriosis, Chen et al. found that the first group had higher values of NLR than the second one (31). In a comparison of NLR between endometriosis patients and healthy controls, Zhou et al. found that the NLR was significantly higher in the first group (36), this result was confirmed also by Turgut et al. (28), and by Kedzia et al. (30).

The role of NLR has also been investigated regarding its capacity for predicting pregnancy success after surgery in women with ovarian endometrioma who desire pregnancy. Findings showed that higher values of NLR were associated with better natural pregnancy outcomes after intervention. The main cause of infertility in this group of women is likely inflammation associated with endometriosis (34). Also, Jing et al. found that an infertile endometriosis group had lower values of NLR and CA 125 than a fertile group (33).

Khashchenko et al. also demonstrated that NLR values were reduced after medical therapy (24). Dominoni et al. showed that NLR is associated with chronic pelvic pain, although this result was not statistically significant (21). Gorun et al. found that NLR was higher in patients with endometriosis and pelvic pain than in those without pelvic pain (29).

Regarding Ca 125, Yavuzcan et al. showed that CA125 levels were higher in patients with endometriosis than in patients with benign ovarian masses (26). These results were confirmed by Cho et al. (22), by Yang et al. (35), Tokmak et al. (25), Jing et al. (33), Din et al. (32) and Kayacik et al. (27). CA 125 is also reduced after therapy (24). Again, as for NLR, Chen et al. found that women with ovarian cancer (not only benign tumors) had higher values of CA 125 than women with endometriosis (31).

In patients with negative CA 125 values, Yang et al. found that NLR and the combination biomarker of NLR and CA 125 had better accuracy in the detection of stage III and IV of endometriosis (35). Turgut et al. found that Ca 125 was significantly higher in more advanced stages of endometriosis (28); there was no difference in NLR and CA 125 between stage III and IV of endometriosis (23), but significant difference was recorded between stage I-II and III-IV (33).

Jing et al. found that the combination of NLR-CA 125 increased the sensitivity and decreased the specificity in differentiating endometriosis from benign ovarian tumors and healthy women compared with CA 125 alone (33).

When Yang et al. compared CA 125 and NLR they found that CA 125 has better sensibility and specificity in diagnosing endometriosis, as confirmed also by Ding et al. (32), by Tokmak et al. (25), and by Kayacik et al. (27). This result is not in agreement with Turgut et al., who found CA 125 has better specificity than NLR, but lower sensitivity than NLR in diagnosing of endometriosis (28). However, NLR seems more able to detect patients with minimal to-mild endometriosis (22). In addition, the combination marker has the best sensibility (32, 35) and specificity (25). This result is confirmed also by Jing et al. (33) and Cho et al. (22). The combination of markers seems to be more effective in diagnosing patients with moderate to severe endometriosis. On the other hand, Cho et al. found that NLR has better sensitivity but lower specificity than Ca125 (22).

Other reviews and meta-analyses have investigated the role of NLR in endometriosis diagnosis. For example, Tabatabaei et al. concluded that NLR levels were higher in endometriosis patients than in healthy women and patients with benign tumors. In addition, they found that NLR did not change significantly among different stages of endometriosis. The pooled sensitivity and specificity of NLR were 0.67 and 0.68, respectively (41). Moreover, Ottolina et al. have studied coagulation and inflammatory markers in endometriosis patients, and the results of their review show two studies that found no difference between cases and controls in terms of NLR, and four studies reported NLR values significantly increased in the endometriosis group compared to benign ovarian tumors and healthy controls (20).

### Limitations

It is important to note that the studies included are heterogeneous. Not all studies considered the phase of menstrual cycle before recording CA 125 values, which is influenced by menstruation. Some studies included a small number of patients. Furthermore, there is no consensus on the cutoff of NLR, and this was calculated differently among the studies. The control group is not homogeneous between studies, because it sometimes included only healthy controls and sometimes also women with benign gynecological diseases. Finally, some papers included adenomyosis as endometriosis manifestation, while others excluded it. As previously declared, there is great heterogeneity among studies.

## Conclusion

There are contrasting results about the non-invasive diagnostic role of both CA 125 and NLR for diagnosing endometriosis. However, our meta-analysis demonstrates the low specificity and sensitivity of NLR. Good outcomes came from studies that investigated the combination of the markers. However, it seems unlikely that the determination of these markers will help substantially in the diagnosis of endometriosis. Assessing NLR and CA125 values, as well as their combination, could be interesting in cases with clinical symptoms of endometriosis, however this assessment is currently not considered crucial. In particular, the determination of NLR lacks specificity and sensitivity, so would be particularly unhelpful in cases in which it is difficult to determine the presence of endometriosis at US or MRI.
