# Supplementary material for: Neutrophil-to-lymphocyte ratio and endometriosis: systematic review and meta-analysis
Source: Front Med (Lausanne). 2026 Jun 22;13:1813357. doi: 10.3389/fmed.2026.1813357 (PMC13333614; doi:10.3389/fmed.2026.1813357)
Supplement: Supplementary file 1 [file Table_1.DOCX]

| **Database** | **Search query** | **Search database** | **RESULTS** |
| --- | --- | --- | --- |
| **Pubmed** | ("Endometriosis"[Mesh] OR endometriosis[tiab]) AND ( "neutrophil to lymphocyte ratio"[tiab] OR "neutrophil-to-lymphocyte ratio"[tiab] OR NLR[tiab] OR ("Neutrophils"[Mesh] AND "Lymphocytes"[Mesh]) OR (neutrophil*[tiab] AND lymphocyte*[tiab]) ) | Filters: English, from from 2008/1/1 - 2025/5/31 | **73** |
| **Embase** | ('endometriosis'/exp OR 'endometriosis' OR 'endometriosis':ti,ab,kw) AND ('neutrophil to lymphocyte ratio':ti,ab,kw OR 'neutrophil-to-lymphocyte ratio':ti,ab,kw OR 'nlr':ti,ab,kw OR (('neutrophil'/exp OR 'neutrophil') AND ('lymphocyte'/exp OR 'lymphocyte')) OR ('neutrophil*':ti,ab,kw AND 'lymphocyte*':ti,ab,kw)) | AND [english]/lim AND [01-01-2008]/sd NOT [01-06-2025]/sd AND ('article'/it OR 'editorial'/it OR 'letter'/it OR 'review'/it) | **184** |
| **Web of Science** | (TS=(( endometriosis ))) AND TS=(( "neutrophil to lymphocyte ratio" OR "neutrophil-to-lymphocyte ratio" OR ("NLR" AND neutrophil*) OR (neutrophil* AND lymphocyte*) )) | Index data: 01/01/2008 to 2025-05-31 .Database: Web of Science Core Collection, Grants Index, KCI-Korean Journal Database, MEDLINE® , ProQuest ™ Dissertations & Theses Citation Index, SciELO Citation Index; Language: English | **74** |
