# Supplementary material for: Neutrophil-to-lymphocyte ratio and endometriosis: systematic review and meta-analysis
Source: Front Med (Lausanne). 2026 Jun 22;13:1813357. doi: 10.3389/fmed.2026.1813357 (PMC13333614; doi:10.3389/fmed.2026.1813357)
Supplement: Supplementary file 2 [file Table_2.DOC]

S1: Quality of cross sectional studies inserted in the analysis

|  | Study sample selection | | Assessment of exposure and outcomes | | Confounding factors | | Total |
| --- | --- | --- | --- | --- | --- | --- | --- |
| Study | Representativeness of the study sample | Sample size | Assessment of exposure | Assessment of the outcomes | Adjustment for confounders | Assessment of confounders |  |
| Zhou et al, 2025 [36] |  |  | * | * | * | * | 4 (Moderate) |
| Turgut et al., 2019 [28] |  | * | * | * | * | * | 5 (Moderate) |
| Chao et al, 2008 [22] |  | * | * | * | * |  | 4 (Moderate) |
| Ding et al., 2019 [32] |  | * | * | * | * |  | 4 (Moderate) |
| Jing et al., 2020 [33] |  |  | * | * | * | * | 4 (Moderate)) |
| Tokmak., 2016 [25] |  |  | * | * | * | * | 4 (Moderate)) |
| Yang et al., 2013 [35] |  |  | * | * | * | * | 4 (Moderate) |

Legend: asterisk represents the satisfaction of the specific item.
